# Choreographing Triage: Making Patient Requests ‘Flow’ Through Digitally Enabled Systems of Access and Decision‐Making in NHS Primary Care

**DOI:** 10.1111/1467-9566.70172

**Published:** 2026-04-02

**Authors:** Natassia Brenman, Sophie Spitters, Sharon Spooner, Jospeh Wherton, Michael Gill, Deborah Swinglehurst, Sara Paparini, Sara Shaw

**Affiliations:** ^1^ Nuffield Department of Primary Care Health Sciences University of Oxford Oxford UK; ^2^ Health Services Management Centre University of Birmingham Birmingham UK; ^3^ Division of Population Health Health Services Research & Primary Care University of Manchester Manchester UK; ^4^ Said Business School University of Oxford Oxford UK; ^5^ Wolfson Institute of Population Health Queen Mary University of London London UK

## Abstract

This paper explores the altered landscape of primary care following the accelerated process of implementing digitally enabled triage across UK general practice in response to COVID‐19. Traditional understandings of triage are based on a static ‘pile sorting’ logic, which suggests that triage outcomes depend upon a single decision in time and space. With the introduction of remote, asynchronous and distributed decision‐making, triage needs a more dynamic conceptualisation. Drawing on a team ethnography in three GP practices in England, we develop the concept of triage choreography to explore the (often hidden) work involved in achieving the flow of patient requests through triage systems. We ask who participates in this work, who might be excluded and the consequences for triage outcomes. Our findings extend the literature on digital in/exclusion in primary care, providing a critical analysis of ‘flow’ in digitally enabled triage and what it means for patients. We show how, as patient requests enter digital systems, triaging work becomes distributed, often in uneven ways. And although digitally enabled routes through systems afford faster and smoother movement, they can also limit patients' ability to influence how they access care and the modality in which it is delivered.

## 1 Introduction and Background

On a Monday morning in an inner‐city English GP practice, the consultation slots are filling up fast, but the waiting room is remarkably quiet. It is a stark contrast to the prepandemic period when patients could book appointments in person, and they even ran a regular walk‐in triage session. Now, if patients do walk into the surgery, the first thing they see is a whiteboard in front of the reception desk informing them that ‘from April 2021, in response to patient demand, we will be using online patient triage to support our patients getting help in a timely manner’. This digitally enabled triage system promises faster, fairer and more efficient access to GP care. The hastily written sign is a reminder of the rapid change and adaptation that took place in this GP practice (and across primary care) in response to the COVID‐19 pandemic. The fact that, 2 years later, it remained propped up on the same tripod was a sign of the continued need to respond urgently to high demand and evolving pressures on the GP practice.

(Metro Group Practice, Spring 2023)

This ethnographic vignette is taken from our study of decision‐making in primary care triage. It illustrates how the landscape of primary healthcare access in the United Kingdom was profoundly reshaped by the rapid shift towards digital and remote care technologies during the COVID‐19 pandemic (Murphy et al. 2021; Greenhalgh et al. 2022). Most GP practices returned to delivering in‐person appointments while strongly encouraging the use of telephone, text message and (occasionally) video consulting. We refer to these different appointment types as ‘mode of consultation’. The contemporary primary care landscape is thus characterised by hybrid models of remote and in‐person care (Chappell et al. 2023), making the question of how to access care (the mode of access) and who gets what kind of care (the mode of consultation) much more complex.

Primary care triage systems aim to add structure to this complexity, incorporating decision‐making about mode of consultation as well as urgency, data gathering and managing workflow. Improving the flow of patients through the system has long been a motivator for introducing triage into healthcare settings (Harding et al. 2011). This became an imperative in primary care during the pandemic, with NHS guidance urging practices to ‘manage demand through a single workflow, prioritising care based on need’ (NHS England 2020). Central to this strategy were the remote and digital aspects of triaging, which have remained a priority in the vision of modern general practice access that was set out for the NHS in 2023 (NHS England 2023). Foregrounding the use of digital tools such as online consultations,^1^ the emphasis has been on flow and efficiency as a means of getting patients to the right healthcare service in the appropriate time frame.

Three recent developments are critical here. First, the *remote* elements of digital triage platforms add another dimension to care and agency ‘at a distance’ (Mort et al. 2003; Pols 2012), as patients are encouraged to express needs and preferences remotely and asynchronously before they reach the clinical encounter (Chappell et al. 2023). Secondly, the ‘online consultation platforms’ that provide digital access require patients to use particular capabilities and skills to create a ‘digital facsimile’ of themselves to convey their problem (Dakin et al. 2024). Finally, the deployment of support staff to receive, filter and ‘pre‐triage’ requests across a wide range of potential clinical roles^2^ has brought renewed relevance to Rapley's notion of *distributed decision‐making* in clinical practice (Rapley 2008) to contemporary primary care triage.

Absent from the conversation to date is a critical analysis of the movement and ‘flow’ of patients through contemporary primary care triage. In the context of digitally enabled triage, the flow of patients becomes the *workflow of patient requests:* the multitude of patient needs which must be organised into appropriate care encounters. The introduction of digitally enabled triage organised around a single workflow has been proposed as a key strategy to tackle the continued build‐up of unmet need in primary care since the pandemic (NHS England 2023). To date, the constituent parts of triage systems have been critically analysed (Marshall et al. 2018; Turner et al. 2022; Dakin et al. 2024), but limited attention has been paid to *triage workflows.* Rather than taking the efficient flow of patient requests as an automatic and positive outcome of implementing digital triage, we are interested in the ‘hidden work’ (Barnard et al. 2024) and sociotechnical arrangements (Berg 1999) involved in creating this movement.

In this paper, we ask: *What sociotechnical work makes patient requests* ‘*flow*’ *through the triage system? Who participates in this process, when and where*? *With what consequences*? Using an ethnographic approach, we examine digitally enabled triage systems as a still‐emerging space of care (Schillmeier and Domènech 2010), characterised by *movements towards* various remote and/or in‐person clinical encounters, extending the longstanding conversation on the dynamics of ‘care at a distance’ (Pols 2012). As is well established in the sociotechnical literature, healthcare practices are part of heterogeneous networks that come together to create smooth functioning (Berg 1999). Similarly, in contemporary sociological approaches to digital health, technologies do not function in deterministic ways but rather are dependent on sociomaterial practices (Henwood and Marent 2019). It is therefore important to unpack taken‐for‐granted assumptions about what digital triage technologies might achieve *in and of themselves* and consider the complex configurations of people, places and technologies needed to achieve smooth flow and coordinated decision‐making.

To do this, we turn to choreography, extending the way this concept has been used in medical sociology, anthropology and STS to theorise primary care triage as a collaborative, distributed achievement. In the sections below, we first develop the concept of ‘triage choreography’ before setting out our methods and ethnographic findings. Our findings contribute to the social science literature on the spatial and temporal dynamics of triage by providing a critical analysis of ‘flow’ in digitally enabled triage and what it means for patients. In doing so, we advance understandings of triage as movement in an increasingly digitalised primary care context and uncover the hidden work of achieving this movement.

### 1.1 Triage Choreography: Theorising New Spatial and Temporal Arrangements of Triage

We advance the notion of *choreography* to make sense of the new spatial and temporal arrangements involved in triage in an increasingly digitalised system of care (Oudshoorn 2011). The term choreography is used here as a metaphor to describe these dynamic arrangements of access and triage, such as patient flow. Originating from the context of dance, it is a device to analyse movement, invoking questions around discipline, improvisation, materiality and performance. Its use in the social sciences can be traced back to Goffman (1959). We define *triage choreography* as the spatiotemporal coordination of the flow of patient requests towards the ‘right’ appointment type with the ‘right’ person at the ‘right’ time. This draws on two related concepts: (1) the spatiotemporal choreography of creating smooth ‘flow’ through contemporary triage systems and (2) the ontological choreography (Cussins 1996) of achieving the ‘right’ triage decision. The first takes inspiration from scholarship on the ‘choreography’ of physical movements, flows or containment within clinical spaces (e.g., Brown et al. 2021) and ‘careography’, a sociospatial concept that highlights how clinical decision‐making is ‘staged, timed and coordinated by medical staff’ (Navne and Svendsen 2018a, S560). The second concept—ontological choreography—is a device to understand how technologies and social relations are coordinated to achieve certain phenomena. Charris Cussins developed this analytic concept in her ethnography of infertility clinics, describing the work of ‘making parents’ through a ‘complex choreography of nature, society, bodies, selves, and technologies’ (Cussins 1996, 8). The concept has been extended to care practices and bureaucratic apparatus, for example, in assisted dying, where ‘dying people and caregivers to jointly produce and stage manage death’ (Buchbinder 2018, 485), with Lewis et al. (2024) highlighting the collaborative and partially improvised nature of this kind of choreography. These accounts emphasise the role of technologies as ‘moving parts’ within wider social and institutional dynamics, meaning they are rarely simple nor neutral.

Our conceptualisation of *triage choreography* acknowledges that triage has continued to evolve beyond its original function as a rapid process of delivering emergency medicine on the battlefields of the Napoleonic wars (Mitchell 2008; Solomon 2017). Medical decisions were once singular moments of (literally) sorting bodies in time and space. But triage has continued to change, encompassing an evolving range of actors and shifts in the regulatory and policy context over time (Andersen and Aarhus 2019). The way we think about triage has also changed: Some social scientists have critiqued triage logics, drawing attention to the ethical and epistemic stakes of these ‘sorting’ practices (Bowker and Star 1999; Redfield 2013), whereas others have considered triage as an extended set of practices rather than single points of decision‐making. For example, Solomon (2017, 350) has described triage as an ‘iterative, spatial process’. As we go on to explain, our notion of contemporary triage as choreography helps us understand the increasingly digitalised process of triage in ways that previous conceptualisations have not considered. For instance, by illuminating the different dynamics involved in coordinating the additional technological and social ‘moving parts’ that online consultation platforms bring to the triage process.

We argue that defining triage choreography in terms of its spatiotemporal *and* ontological aspects allows us to understand the relationship between process and outcome in digitally enabled triage. In other words, how patient requests move through distributed systems and how this shapes participation in triage decision‐making. This approach highlights an important paradox: Although digitally enabled triage expands the (spatial, temporal and relational) complexity of access and decision‐making, the model of a ‘single digital workflow’ for triage renders many of these moving parts invisible. This invisibility can make it harder for some actors than others to participate in the process of crafting a good or right decision about care. Surfacing and interrogating these moving parts enabled the generation of novel insights into (digital) inclusion and exclusion in contemporary primary care beyond single points of triage decision‐making.

## 2 Field Sites and Methodology

This paper draws on ethnographic data from the ‘ModCons study’ (Mode of Consultation in General Practice), funded by the National Institute of Health Research School for Primary Care Research (2022–2025), examining decision‐making about who gets what kind of care, with a focus on remote or in‐person modalities. This was a substudy of the Remote‐by‐Default 2 (RbD2) project (Greenhalgh et al. 2025), which sought to inform safe and equitable care in UK general practice in the context of policies requiring phone, video or online consultation tools ‘by default’. All patients and staff interviewed and/or observed in clinical consultations gave written or verbal informed consent in accordance with our ethics protocol.

We conducted a team ethnography (Bikker et al. 2017) across three case study sites to understand how, when, by whom and why decisions are made to offer these different types of appointment. Our three case study sites were selected from a total of twelve Remote‐by‐Default 2 study sites. The inner‐city Metro Group Practice, which served approximately 16,000 patients, was the most digitally mature and operated a total triage system, with senior clinicians triaging all patient requests via a single digital workflow. The semi‐rural Green Park Group served approximately 15,000 patients from multiple villages and rural communities across two sites. A total triage booking system was introduced shortly before our fieldwork began, using two different digital triage platforms (both company names pseudonymised throughout). The urban Red Brick Practice served 30,000 patients across three sites and, in contrast to the other sites, ran a receptionist‐led hybrid triage model (described in more detail below).

Fieldwork took place over 15 months (2022–24). An initial familiarisation phase in each practice was followed by two separate intensive data collection phases in three sites, each involving fieldwork with between 2–5 ethnographers present from 8 am to 6 pm throughout the working week. We tracked consulting activity in each practice over a total of six weeks, observing and interviewing clinicians, support staff and patients. In total, we conducted approximately 600 h of observations, involving a range of (clinical and nonclinical) staff engaged in all aspects of access and triage, following some cases through to appointments and follow‐up care. We completed 15 ‘sit down’ interviews and 30 ‘go‐along’ interviews (Garcia et al. 2012) with practice staff focusing on the formal, informal and ‘invisible’ work (Star and Strauss 1999) involved in making decisions about consultation type. Finally, we produced in‐depth narratives of patients with complex care needs, based on 20 interviews and reviews of appointment histories over 24 months.

We developed our analysis of triage work as we enriched these more traditional ethnographic methods with a novel method to track the trajectories of patient requests. We produced process maps of triage and appointment booking systems to understand planned, structural aspects of decision‐making; then, we observed and tracked how they played out in practice (Feldman and Pentland 2003). Mirroring the collaborative, distributed decision‐making practices we had already started to observe, we positioned our team across 2–3 key points in the triage process, communicating and coordinating our observations in real time on a (secure) shared document. We generated ethnographic field notes, maps and summary tables that tracked the trajectories of patient requests. Initial analysis of these data sources provoked us to look to the concept of choreography to further sensitise us to the dynamic movement we were observing in the data. The lead author continued to generate initial themes (Morriss 2024), theoretically informed by literature using choreography to describe spatial dynamics of care (Pinto 2013; e.g., Brown et al. 2021), as well as the broader sociotechnical literature that informed our research question (Berg 1999). We iteratively developed and refined our use of theory as we progressed with the analysis, for example, using the notion of ontological choreography to understand triage technologies within wider social, ethical and institutional dynamics. The initial themes formed the basis of several data workshops in which the ethnography team refined and developed the themes as ‘patterns of shared meaning’ (Braun and Clarke 2019) around the flow of information, work and patients and practices of coordination and improvisation in triage, which we describe below.

## 3 Ethnographic Findings

### 3.1 Optimising Flow

In this section, we introduce how the goals to optimise the flow of patient requests in policies and protocols were put into practice in our case study sites, focusing on the social, material and technical infrastructure that supported this. In addition to the digital workflows built into triage systems (described above), we were aware of ongoing efforts within organisational and national policies to tackle the build‐up and congestion of patients within triage and appointment‐booking systems. For example, during our fieldwork, a change was made to the GP contract which stipulated that ‘all patients should be offered an assessment of need, or signposted to an appropriate service, at first contact with the practice’ (Kaffash 2023). Below, we trace how these imperatives translated into local practices and discourse across the three sites. The crafting and coordination of what practice staff call ‘smooth’, ‘quick’ or ‘efficient’ flow is a key part of the spatiotemporal choreography of triage.

#### 3.1.1 Organising the Patient Flow

Many of the organisational documents supplied by practice staff and our early conversations with GP partners mirrored the emphasis on flow, streamlining and efficiency that can be found in NHS policies. Staff members showed us flow charts and tables that illustrated carefully thought‐through processes, with decision trees to be shared with the range of practice staff now involved in booking appointments. In the Green Park Group Practice, where triage decisions were most widely distributed across people and technologies, the notion of *smooth flow* emerged as a distinct value within the talk and work of the team. One member of the reception team kept coming back to his role in creating ‘smoothness’ in the system. His job was to filter incoming requests and booked appointments, and he had various tricks to optimise the infrastructure around triage, such as maintaining a quick turnover of requests and ensuring they were all in the correct digital form for review by the triage doctor. Although technology was often used to create or optimise smooth movement, this was not always the case. The Red Brick Practice had abandoned a telephone‐based total triage system in favour of the more low‐tech solution of upskilling reception staff to ‘organise the patient flow’ (in the words of the practice manager), drawing on formal and ad hoc training as well as ‘common sense’ (in the words of reception staff). All three sites preserved some aspects of the spatial and material ‘choreographies of social distancing’ (Brown et al. 2021, 200) put in place during the pandemic: Clear waiting rooms, Perspex screens and visual prompts to use online systems for appointments and prescriptions. This enabled (and concealed) much of the digital and nondigital work that reception staff did to create and maintain the flow of access and triage behind the front desk.

We observed a wide range of offline practices and arrangements that needed to be in place to organise the flow of requests, even in the practice which was most focused on using technology to optimise flow and efficient access. The Metro Group Practice was an early adopter of ‘total triage’ (the system of gathering information and making decisions about care before any appointment is booked) and the digital technology designed to support this. As the opening vignette described, the ‘GP Linx’ triage system had replaced the prepandemic walk‐in triage system, something we were told (by staff and patients) was sorely missed by many patients. But the GP partners insisted that the new system provided broader, fairer and faster access overall. Patients were therefore steered away from requesting appointments in person and encouraged to fill out an online form at home. Alternatively, one of the reception staff could fill it out for them over the phone or at reception. All requests were forwarded to the inbox of a triage doctor (two doctors on a Monday morning), who could handle more than 300 requests per day. Even with the help of a second triage GP and the backup of a duty doctor, the most highly trained GPs struggled with this intensity. Although the digital system had radically streamlined the patients' route from access to appointment, the ‘clutter’ that remained in the clinical space had an important role in supporting this system: the whiteboard in the waiting room, staff areas piled high with paper medical records awaiting digitisation and two busy kitchens containing extensive coffee‐making equipment to enable ad hoc discussion about triage dilemmas. This ad hoc collaboration was eventually formalised when a dedicated ‘triage room’ was created from unused waiting room space to support decision‐making among triage doctors, trainees, clinical pharmacists and the duty doctor. And so, ‘digitally enabled triage’ was *also* enabled by a distinct set of offline practices, relationships and spatial arrangements.

#### 3.1.2 Digitising Patient Requests

Choices about the design and adoption of primary care triage systems suggest that patient requests do not simply ‘flow’ like water; they are *made to move* in particular ways by multiple human and nonhuman actors. Two of our field sites (the Metro Group Practice and Green Park Group Practice) had digitally enabled total triage systems in place. The Red Brick Practice had adopted a hybrid system, led by the receptionist team, which meant that only cases that were particularly complex or uncertain were triaged remotely by a GP. In all three sites, remote requests had to travel through space (via telephone or remote consulting platforms) and often had to be held in a system to enable asynchronous decision‐making (sometimes by multiple staff members). To this end, requests would be digitised to make them amenable to downloading, storing in different software systems, filtering, sorting into categories of urgency and sending between GPs and support staff. In both total triage systems, all requests had to be entered into the single digital workflow to be considered by a triage doctor before any appointment decisions were made. But it mattered *how* information was entered; for example, the Green Park practice always used an in‐house template for information gathering during telephone or walk‐in requests. Reception staff actively sought to maintain the wholeness of each patient and their story as much as possible, and this would help them sort or ‘pre‐triage’ requests into urgent or routine. By contrast, the Metro Group Practice always used the digital format of online requests, regardless of access route. This was designed to get information from the patient to the triage doctor in what they described as a ‘direct line’. This was highly effective in making the requests flow quickly through triage but required patients and staff to accept trade‐offs, such as the triage interface feeling ‘faceless’ to some patients, in the words of one triage doctor, and reflected in some patient interviews.

In addition to managing how information ‘gets onto’ the digital system (Swinglehurst and Greenhalgh 2015), there were also choices to be made about which digital triage platform to use and how. We saw two platforms being used across sites, with one site switching from one to the other during our fieldwork. The Metro Group had always used GP Linx, which elicits written information from patients in free‐text boxes, ready to be reviewed by a triage doctor. The second triage platform, E‐doc, takes patients through a questionnaire to ascertain urgency. Responses can trigger preconfigured risk labels (called ‘red flags’) that can reroute the patient to urgent pathways such as hospital accident and emergency departments before practice staff see the request. As we describe below, the Green Park Group abandoned E‐doc and replaced it with GP Linx due to patient and staff frustration with the lengthy questionnaires. The Red Brick Practice persevered with E‐doc but combined it with a more traditional receptionist‐led booking system. If needed, receptionists could enter more detail about the request into the digital system and send it to a senior doctor who could help decide about urgency and modality. The trade‐off was that although patients could access care via a direct (nondigital) interaction with a receptionist, they would be waiting much longer for an appointment than patients who were able to use the online system as a ‘quick way in’, as the GP partner in charge of patient flow explained it. In sum, Green Park's shift towards GP Linx prioritised clinician input and reduced rule‐based alerts, whereas Red Brick retained E‐doc to preserve the role of receptionists and their ability to judge risk and urgency with the help of technology. Once the requests had been organised in this way, the work of assessing them and directing them to the correct consultation type began—our focus for the next section.

### 3.2 Coordinating the Temporal and Spatial Dynamics of Digital Triage

The work of managing patient requests was distributed across multiple actors and technologies in each practice. In GP‐led triage systems, the responsibility for triage decision‐making was concentrated in the clinical space where the designated triage doctor sat. Conjuring images of an air traffic controller, triage doctors would work surrounded by multiple screens, with multiple windows open in each, taking calls on a telephone headset. They simultaneously dealt with various channels of digital communication, such as internal messaging systems and email, as well as the incoming flow of patient requests onto their triage list. One doctor would habitually kick off her shoes, anchoring herself with crossed stockinged feet under the large desk and managing this ‘control panel’ positioned slightly above her eyeline, whereas another had invested in a gaming chair to manage the physical intensity of long sessions dealing with hundreds of patient requests. Additional references to virtual worlds lie in the way doctors described triage work—‘Tetris’ and ‘4‐dimensional board games’, for example—perhaps a deliberately incongruous metaphor, given their acute awareness of something very real going awry (unlike in a mere game). Despite the imagery of a lone gamer or air traffic controller, triage coordination was a distinctly collaborative and relational process. Triage doctors translated protocols into practice in their own distinct ways, shaped by relationships with reception and back‐office staff, as well as individual patients.

#### 3.2.1 Speeding up and Slowing Down Patient Flow

The *pace* of flow was something that had to be carefully monitored and managed. The most unsurprising aspect of this was generating fast and efficient movement of patient requests. On a Monday morning in the Green Park Group Practice, Dr M explained the importance of attending to the urgent requests first but also referred to ‘the challenge to keep the E‐doc and nonurgent lists ticking on’, particularly as some urgent requests ‘*trickle in*’ to these filtered lists and must be dealt with quickly rather than being misplaced on the priority list. Another triage doctor welcomed the capabilities of their new digital triage system to open a few seconds faster than the old E‐doc software. She also liked the fact that it did not overwhelm her with automatically generated patient risk information (as E‐doc and other rule‐based triage tools can do). Several triage doctors spoke about the need to use their own judgement to actively respond to requests rather than mechanistically working through the triage lists—a quick examination with the ‘on‐call’ doctor for a busy single mum or some online nutrition advice sent to someone with constipation, for example. This reduced the number of appointment slots needed and sped up the overall flow of patients out of the system.

More surprising was the need to *slow down* the flow of requests. We have already described concerns about the slow access granted to those who do not use E‐doc at the Red Brick Practice. In the other two sites, where they operated total triage, the speed of access could be actively managed, but this generated additional work and debate among staff. In the coffee room at the Green Park Group site, Dr M talked about the ‘big debate’ they were having about ‘how easy to make access’, given the introduction of remote care and access routes. Some doctors believed they could maintain a slightly higher threshold of accessibility by favouring in‐person appointments, for which patients need to make the effort to turn up if they really need. Dr M was in favour of this strategy to tackle ‘excess demand’. In his words, ‘It's necessary to put up a barrier for some things. We need to use our time and patients' time strategically’. Before the transition to GP Linx, other members of staff were also concerned that its lack of risk filters (which would flag and redirect some urgent requests) would encourage people to access care online when they should go to urgent or emergency services.

We saw how practice staff slowed the incoming flow of requests when it started to overwhelm their capacity in the Metro Group site. One Monday morning at 9.30 am, they found themselves at full capacity due to a combination of high demand and a scheduling error that meant they had few available urgent slots. As Dr C set about managing this ‘slight disaster’ by mobilising the triage and duty doctors to meet requests at the point of triage, he described the problem of ‘access driven demand’. He suspected that the fact that they offer excellent, quick access might encourage people to expect and ask for more, making the practice constantly vulnerable to reaching the limit of their capacity and becoming overwhelmed. We observed the team managing this as we set about tracking patient requests, with one researcher stationed at reception and another in the triage room upstairs. Considering the request from a patient known to both the reception staff and the triage doctor for her twice‐weekly requests for care, the triage doctor opted *not* to book her into the next available routine slot, instead choosing one later that week. ‘You try to manage these patients by having them wait a bit longer for their appointment—then they can learn to manage their own wellbeing a bit more’. Like Dr M, he was slowing down the access pathway, taking the initiative to put *time* between the patient requests and the consultation itself to minimise ‘access driven demand’.

#### 3.2.2 ‘The Distance Can Be Helpful’

If what we have been describing above is the sociotechnical work of managing the temporal distance between patient request and care, there is also a spatial element to this work. We were made aware of anxieties similar to those about the *speed* of access enabled by digital triage when it came to how *direct* the route to a clinician becomes when patients can send requests to be reviewed by a triage doctor. In total triage systems, this mode of accessing care is set up to bypass the reception staff, who have traditionally been framed as gatekeepers or, as one triage doctor described them, ‘buffers’ between patients and GPs: ‘The receptionist used to be the buffer, so it's a bit stressful for us [triage doctors] now’ (Dr C, the Metro Group Practice). From the perspective of triage doctors, there is what they called a ‘direct line’ between them and the patient when they receive a message in their E‐doc or GP Linx inbox. However, the spatial arrangements of the GP surgeries and the technological infrastructure enabling remote access maintain physical distance between the patient and the triage doctor and mediate the line of communication between them. For example, through the positioning of the triage doctor well away from patients in a consultation room or dedicated triage room, there was an opportunity to glean, discuss and act on information about patient requests without synchronous face‐to‐face interactions. The benefits of this distance for the triage doctor were made visible in a session with Dr M:

An E‐Doc request comes in from a parent who suspects that her child has autism. She has been directed to her GP by a (non‐specified) professional to request an assessment for diagnosis. As Dr M reads this, he immediately opens up a digital text message box to suggest going via the school system, talking as he types:

’Go there first and if there are any issues, feel free to drop us a message to arrange an appointment’. He looks online for signposting resources that will direct the parent to the educational route, emphasising that getting a diagnosis is a long process. Later, we debrief about the use of GP Linx messaging in triage. He says that patients can feel confused or ‘fobbed off’ when they receive a text message in response to an appointment request. On the other hand, he says that ‘the distance can be helpful’.

’In this case’, he says, ‘it is much harder not to pick something like this up if you are face‐to‐face with someone in distress’.

Here, Dr M felt he needed to distance himself from the physical cues of help‐seeking to get the patient to the ‘right place’. The distance helped bypass the lengthy process of taking on the case or the alternative, emotionally taxing work of denying the patient what she wanted in a face‐to‐face encounter.

### 3.3 Improvising Participation in Digital Triage

In this final section, we address the question of who participates in the triage process, with a focus on how, when and where patients are involved. First, we look at how patients navigate and (seek to) influence the digital triage process. We then focus on scenes of in‐person participation in the triage process, drawing on examples of patients showing up in person, bypassing the digitisation of their request in order to shape or disrupt triage decision‐making.

#### 3.3.1 Improvising Digital Negotiation

In response to these new digital channels for appointment requests, we saw patients experimenting with ways of articulating their need and routes through the digital system. Several styles of digitally communicating need were prominent as we observed triage doctors at work in the total triage systems. Most striking were those seeking to express urgency or distress by using the so‐called ‘direct line’ to triage doctors to invoke a particular response. One patient filled in the GP Linx form by writing ‘I NEED TO SEE SOMEONE, I NEED TO SEE SOMEONE …’ repeatedly in capital letters in each field, ignoring the specific requests for information and focusing on the urgency of her (so far unmet) need for a face‐to‐face consultation. Patients also described how they had learnt to encode their need for an urgent appointment or a certain modality (usually, but not always, in‐person) via vocabulary such as ‘severe mental illness’ or ‘complex patient’, or visually, with the photos they could attach to online appointment requests. Triage doctors would, in turn, seek to decode this language, wary of taking shocking expressions of need at face value. One triage doctor responded to a message with graphic descriptions of vomiting blood as ‘testing the boundaries … of whether the doctor would call back’.

The ‘dance’ of reaching out, responding to and negotiating need would only go so far given the parameters of the technology. Patients cannot respond to a triage decision via the same digital channel, unless the doctor requires more information and decides to ‘allow response’. This is an important safety feature of the technology—guarding against conversation threads being left open and unread—but it forecloses the dialogue that patients often seek to successfully negotiate the care they need. Similarly concerning for staff was the use of email for patients’ requests. Email is not linked to the electronic record and cannot be embedded in the triage workflow, so email requests must be individually transferred, putting them at risk of being missed. Exploring these issues in patient interviews helped us understand that such ‘unruly’ practices^3^ often spring from a sense of being unable to navigate the designated triage systems: ‘my fear is I will get lost in the website I'm trying to navigate my way through’. Another older woman described being ‘inventive’, using multiple phone lines to navigate the 8 am queues and avoid using E‐doc where ‘you give your details of what's wrong with you and somebody at the end of the form reads the form and tells you whether you're worth it’. For these patients and many others, the opacity of the digital triage system made participating in decision‐making challenging, and they perceived it essential to improvise in whatever ways seemed to work best.

#### 3.3.2 Disrupting the Flow

In all three practices, it was possible to request help offline, and (even within so‐called ‘total triage’ systems) there were ways to negotiate about urgency and appointment type in a nondigital space, for example, by simply turning up at the GP surgery. In contrast to online channels, this did not require specific technical or literacy skills, meaning it was more straightforward for some patients to shape the pathway to care and triage decision in this way. However, the availability and visibility of this option were different across the three practices. In the Red Brick Practice, which operated a receptionist‐led system, most decisions about care took place around the front desk, providing more possibilities for in‐person dialogue than in total triage systems. This gave visibility to requests for care that may never have reached remote total triage, such as from the older man who spoke limited English we observed struggling to articulate his request to a receptionist. After some minutes of being misunderstood, he eventually just repeated the name of one of the care coordinators, Magdalena, who was originally from his home country. Having translated for him before and knowing his complex medical, mental health and social care needs, Magdalena was able to organise an immediate in‐person appointment with a trainee and follow up with his usual doctor and a social prescriber. Although the practice still encouraged patients to access care using the online consultation tool where possible and the telephone for urgent cases, their system allowed patients to bypass the process of digitising their request, accessing an appointment directly at the front desk.

In the other two practices, in‐person negotiation about appointment type was less straightforward, as all requests were entered into the digital triage system, even when patients sought help in person. In‐person negotiation required patients or support staff to ‘de‐digitise’ the request, usually after the triage doctor had seen it and allocated an (unfavourable) appointment. Staff tended to respond flexibly and effectively, but it required significant patient work to get to this point. We witnessed one such example when shadowing staff at the front desk. A man wheeled his bike into the waiting area ….

Waiting for a member of staff to come off the phone, he jokes that working in a pub everyone used to muck in when it got busy, before batting a hand and saying impatiently, ‘yeah, yeah, I know, things have changed …’ Dee, one of the newest members of reception staff pops out from behind the desk having heard his voice—she seems to know what’s going on. She talks to him in a hushed voice and he immediately stops joking and tells her about a problem with his prescription. He’s been allocated a telephone appointment to discuss this with the GP but he is not happy with the decision: ‘can’t I at least have a real [in‐person] appointment? I’m getting stressed and when I’m stressed, I go off the phone’ he waves a small mobile over his head—a kind of warning shot. After some negotiation, Dee takes a deep breath and says they can try and rearrange the appointment, popping back behind the Perspex screen. It turns out the telephone appointment is scheduled for that afternoon so, by chance, the GP may be able to see him in person instead of over the phone. Dee runs upstairs, saying firmly ‘wait there’. Before long the patient is called upstairs and comes out after 10 minutes with the GP, having a good‐natured chat about next steps. The man presses his hands together towards to Dee as he leaves saying ‘thanks, thanks so much, you are a star’.

Here, the patient managed to momentarily participate in the triage process, disrupting the flow of information and influencing the outcome of the final decision. This was a striking example which stood out because of the work it took to shift the decision‐making course, including administrative, emotional and performative labour from the patient and support staff. The requirement for patients to actively participate in the work of triage was often heightened and could become exclusionary when they wanted to improvise or change the standard process. Crucially, all the practices we work with *did* maintain spaces for nondigital participation in the triage process (with varying degrees of flexibility in the pathways, they have set up). However, creating and maintaining these spaces for negotiation also came at a cost to staff, who were anxious about losing control of the flow of patients and the fair distribution of time, care and resources.

## 4 Discussion and Conclusions

### 4.1 Summary of Contributions

Our ethnographic findings describe the sociotechnical work required to make patient requests move through digitally enabled triage systems towards the ‘right’ appointment type with the ‘right’ person at the ‘right’ time. We contribute to the social science literature on the spatial and temporal dynamics of triage (Andersen and Aarhus 2019; Solomon 2017) and offer a novel, critical analysis of patient ‘flow’ through digitally enabled triage. To do this, we developed the analytic concept of triage choreography, drawing on existing work on the spatiotemporal choreography of care (Navne and Svendsen 2018b; Brown et al. 2021) and ontological choreography in broader biomedical contexts (Cussins 1996; Buchbinder 2018; Lewis et al. 2024). Applying this to contemporary primary care, we were able to surface the complex coordination of patient requests through systems of digitally enabled triage. Without explicitly attending to the coordination of ‘moving parts’, much of this practical and ethical complexity remains invisible, reflecting traditional understandings of triage as single points of decision‐making. Moreover, the introduction of digital systems to create a ‘single workflow’ (NHS England 2020) can suggest that simply introducing these systems will achieve smooth and simple primary care access. In this paper, we demonstrated that the ‘flow’ of patient requests was far from an automatic function of the technology. Through the triage choreography, requests were *made to move* in certain ways that were dependent on sociotechnical and material arrangements of the clinic, as well as wider institutional dynamics and system pressures. Although flow was necessary (to beat the infamous telephone queues and ‘8 am rush’), it was not an unproblematic good, introducing new patterns of in/exclusion for patients.

We extend the idea that there can be multiple actors involved in the crafting, production and management within the ‘choreography’ of clinical care (Buchbinder 2018; Navne and Svendsen 2018b; Lewis et al. 2024). Specifically, we describe the particular and sometimes unequal roles that different actors took in coordinating the movement (or containment) of patient requests. We saw opportunities for staff, but not patients, to adjust the pace and spatial arrangements of the choreography. For example, speeding up and slowing down the flow of requests and the harnessing of distance and technology as ‘buffers’ to mediate negotiations about appointment type. We also found that the smoothness and invisibility of digital triage flows made it hard for some patients to influence how they access care and the modality in which it is delivered. This was true, despite the work required of patients to navigate their way into and through digital systems.

### 4.2 Comparisons With Existing Literature

Our findings about the consequences of (non‐)participation in the choreography of triage corroborate the growing body of research that points to the potential widening of inequalities in increasingly digitalised primary care contexts (Marshall et al. 2018; Dixon et al. 2022). This sits in tension with research that suggests that digitally enabled triage is necessary for the equitable allocation of primary care resources (Rodrigues et al. 2022) and has the potential to *reduce* disparities in access to care (Ge et al. 2024). Service providers in our study were motivated to use triage to improve equitable access to care but found that this disadvantaged some patients in ‘total triage’ systems, creating two tiers of access when it was used in combination with traditional systems. The result is a tension within digital triage: Managing demand within a single digital workflow is designed to achieve broad equitable access under conditions of scarcity, but it also has the potential to produce new patterns of exclusion. This builds on recent work by Dakin et al. (2024) on digital candidacy and Rybczynska‐Bunt et al. (2024) on fractured reflexivity in the digital age, which shines a light on the ways in which negotiation and self‐advocacy can be constrained by triage technologies. Complementing this work on the (often unequal) abilities patients have to *directly* affect triage outcomes, our findings draw attention to the possibilities and limits of patient participation in shaping and affecting the decision‐making *process.* Our analysis of triage choreography suggests that the digitalisation of triage can limit some patients' participation when they reach out, respond to and negotiate different types of care.

Our analysis also highlights uneven possibilities for improvisation in triage choreography. Although improvisation and choreography may appear incongruent, Whalen et al. (2002) developed the notion of ‘improvisational choreography’ to describe how work performances emerge from a combination of spontaneous actions and carefully arranged means and materials. Navne and Svendsen (2018b) also emphasise improvisation in their analysis of ‘careography’, in relation to staff experiences of navigating the tension between exercising medical authority and enabling patient/carer involvement in clinical decisions. Improvisation is commonly described in choreographies of care and speaks to the tensions between plans of action (the ‘dance’) and practices (of the ‘dancers’), particularly in relation to the ‘unanticipated contingencies’ that come with ill health or the end of life (Lewis et al. 2024). This explicitly blurs the boundaries between who or what orchestrates care encounters—in our case, triage doctors and technologies—and those involved in enacting them in practice. We extend this conversation by pointing out the unequal abilities different actors have to flexibly improvise within the triage choreography. Specifically, we describe how patients struggled more than staff to navigate imperfect systems with the (sometimes limited) tools for improvisation available to them. For patients, improvised participation (digital or otherwise) was an important part of making triage work in practice but often came at a cost. It sometimes required breaking the smooth flow of online triage, risking being seen as disruptive or a ‘timewaster’, which most patients we spoke to went to great lengths to avoid. To participate, then, patients must ‘creatively make do with the situation and technologies at hand’ (Nielsen and Langstrup 2018, 259), which can be limited and far from ideal.

### 4.3 Limitations

Our case study sites, although varied in geographical context and patient population, were all relatively innovative, digitally mature and well staffed. Although generalisability is not a goal of ethnographic case study methodology, it is worth considering the implications of these particularities. They contrasted with the ‘deep end’ practices within the wider study we were embedded in (Greenhalgh et al. 2022) and should be considered within the wider landscape of primary care providers subject to modern general practice access policies (NHS England 2023). The fact that they *were* coping with the demand for care meant that they could use digital systems to flexibly respond to challenges and patient needs. Some practices may not have access or capacity to use digital technologies in this way. More significantly, there will be others under such pressure that they are unable to implement new triage technologies while also maintaining the inclusivity of traditional access routes. The hybrid triage system in the Red Brick case study site was arguably the most inclusive; however, as demand increased, a two‐tier system emerged whereby patients accessing care digitally experienced more efficiency and continuity than those seeking care directly over the phone or in person (an example of what Veinot et al. (2018) have termed intervention‐generated inequalities). And so, although our findings point to the benefits of maintaining direct or in‐person access routes, this could paradoxically *increase* inequalities if digital systems are introduced in contexts with less capacity for flexibility.

### 4.4 Conclusion

Developing the notion of triage choreography has allowed us to surface novel insights into the relational, spatial and temporal dynamics of digitally enabled triage, contributing an analytical lens through which to understand who shapes triage processes, when and with what consequences. Although we attend to digitally enabled triage, we demonstrate how primary care triage and the achievement of ‘flow’ are *also* dependent on a physical choreography of material arrangements, facilitating certain kinds of movement, redirection or waiting. We identified multiple ‘choreographers’ making role‐related contributions to the 'performance' of triage. However, the patient can easily disappear into the flow of this triage choreography, particularly as their clinical information is streamlined and standardised in a digital format. The work required from patients to affect how they access care and in which modality is also rendered invisible in these moments, whereas the role of the technology in enabling access is foregrounded. Attending to this patient work surfaced new patterns of exclusion when it comes to who has a say in triage decision‐making, even as providers seek to implement a fairer, more systematic approach to managing demand and access to care. Understanding triage as choreography opens up flexible ways of thinking about and responding to these changing dynamics of digital in/exclusion in primary care access and triage.

## Author Contributions

**Natassia Brenman:** conceptualization, formal analysis, investigation, methodology, project administration, writing – original draft, writing – review and editing. **Sophie Spitters:** conceptualization, formal analysis, investigation, methodology, project administration, writing – review and editing. **Sharon Spooner:** funding acquisition, conceptualization, supervision, writing – review and editing. **Jospeh Wherton:** conceptualization, investigation, formal analysis. **Michael Gill:** funding acquisition, investigation, writing – review and editing. **Deborah Swinglehurst:** funding acquisition, supervision, conceptualization, writing – review and editing. **Sara Paparini:** funding acquisition, supervision, conceptualization. **Sara Shaw:** funding acquisition, conceptualization, supervision, formal analysis, investigation, methodology, writing – review and editing.

## Ethics Statement

The study received ethical approval from East Midlands—Leicester South Research Ethics Committee and UK Health Research Authority (September 2021, 21/EM/0170).

## Consent

All patients and staff interviewed and/or observed in clinical consultations gave written or verbal informed consent in accordance with our ethics protocol.

## Conflicts of Interest

The authors declare no conflicts of interest.

## Data Availability

The data that support the findings of this study are available upon request from the corresponding author. The data are not publicly available due to privacy or ethical restrictions.

## Endnotes

^1^
Turner et al. (2022, e‐128) describe these succinctly as online tools which ‘allow patients to remotely and asynchronously contact a GP using a computer, smartphone or tablet to ask questions and describe symptoms in writing’. Two such tools were used in our case study sites, which we refer to with pseudonymised company names.

^2^
See the Additional Roles Reimbursement Scheme (ARRS) (NHS England 2019).

^3^
In exceptional cases, specific workarounds were agreed upon. One Deaf patient at the Metro Group Practice eventually (after much negotiation) agreed with the practice that he was allowed to email his personal doctor with nonurgent issues, because he felt restricted by the word count in online forms, which was his only option for negotiating with the GP surgery without scheduling an interpreter or doing complicated lip‐reading during consultations.
